# Immune Response Dynamics and Biomarkers in COVID-19 Patients

**DOI:** 10.3390/ijms25126427

**Published:** 2024-06-11

**Authors:** Maral Ranjbar, Ruth P. Cusack, Christiane E. Whetstone, Danica L. Brister, Jennifer Wattie, Lesley Wiltshire, Nadia Alsaji, Jennifer Le Roux, Eric Cheng, Thivya Srinathan, Terence Ho, Roma Sehmi, Paul M. O’Byrne, Maryonne Snow-Smith, Michelle Makiya, Amy D. Klion, MyLinh Duong, Gail M. Gauvreau

**Affiliations:** 1 Division of Respirology, Department of Medicine, McMaster University, Hamilton, ON L8N 3Z5, Canada; ranjbm1@mcmaster.ca (M.R.); cusackruth@hotmail.com (R.P.C.); whetstoc@mcmaster.ca (C.E.W.); bristerd@mcmaster.ca (D.L.B.); wattiej@mcmaster.ca (J.W.); wiltshi@mcmaster.ca (L.W.); alsajin@mcmaster.ca (N.A.); hot4@mcmaster.ca (T.H.); sehmir@mcmaster.ca (R.S.); obyrnep@mcmaster.ca (P.M.O.); duongmy@mcmaster.ca (M.D.); 2 Hamilton Health Sciences, Hamilton, ON L8N 3Z5, Canada; lerouxj@hhsc.ca; 3 St. Joseph’s Healthcare Hamilton, Hamilton, ON L8N 4A6, Canada; eric.sp.cheng@gmail.com (E.C.); thivya.srinathan@medportal.ca (T.S.); 4 The Research Institute of St. Joe’s Hamilton, Firestone Institute for Respiratory Health, St. Joseph’s Healthcare Hamilton, Hamilton, ON L8N 4A6, Canada; 5 Laboratory of Parasitic Diseases, National Institute of Allergy and Infectious Diseases, National Institutes of Health, Bethesda, MD 20892, USA; maryonne.snow-smith@nih.gov (M.S.-S.); makiyam@niaid.nih.gov (M.M.); amy.klion@nih.gov (A.D.K.); 6 Population Health Research Institute, McMaster University, Hamilton, ON L8N 3Z5, Canada

**Keywords:** COVID-19 patients, complete blood count, biomarkers, eosinophils, eosinophil granule proteins, cytokines, chemokines, alarmin cytokines

## Abstract

Background: The immune response dynamics in COVID-19 patients remain a subject of intense investigation due to their implications for disease severity and treatment outcomes. We examined changes in leukocyte levels, eosinophil activity, and cytokine profiles in patients hospitalized with COVID-19. Methods: Serum samples were collected within the first 10 days of hospitalization/confirmed infection and analyzed for eosinophil granule proteins (EGP) and cytokines. Information from medical records including comorbidities, clinical symptoms, medications, and complete blood counts were collected at the time of admission, during hospitalization and at follow up approximately 3 months later. Results: Serum levels of eotaxin, type 1 and type 2 cytokines, and alarmin cytokines were elevated in COVID-19 patients, highlighting the heightened immune response (*p* < 0.05). However, COVID-19 patients exhibited lower levels of eosinophils and eosinophil degranulation products compared to hospitalized controls (*p* < 0.05). Leukocyte counts increased consistently from admission to follow-up, indicative of recovery. Conclusion: Attenuated eosinophil activity alongside elevated chemokine and cytokine levels during active infection, highlights the complex interplay of immune mediators in the pathogenesis COVID-19 and underscores the need for further investigation into immune biomarkers and treatment strategies.

## 1. Introduction

The pandemic of COVID-19 has had profound impacts on global health systems and economies prompting ongoing research efforts to better understand disease pathophysiology and inform effective interventions. Stemming from the novel coronavirus SARS-CoV-2, COVID-19 manifests as an acute respiratory illness, exhibiting a spectrum of severity ranging from mild to severe [1]. Despite considerable progress, our understanding of the immune response in this infection remains incomplete.

Upon the invasion of viruses into the pulmonary system, epithelial cells, serving as the primary line of defense, activate pattern recognition receptors such as nod-like receptors, toll-like receptors, and RIG-1. Upon recognition of viral antigens, these receptors trigger the release of critical signaling molecules including cytokines, chemokines, and alarmins that play a pivotal role in orchestrating the recruitment and activation of various immune cells to mount an immune response against the invading pathogen. The innate immune system relies on the coordinated efforts of cells such as neutrophils, macrophages, and natural killer (NK) cells, each with specialized functions. Neutrophils and macrophages excel in engulfing and eliminating viral particles through phagocytosis, while NK cells specialize in directly targeting and destroying virus-infected cells through cytotoxic activities. In contrast, the adaptive immune response is governed by T helper and B cells, working to orchestrate a tailored immune response against the invading virus. The crucial role of CD8 T cells lies in their ability to recognize and eliminate virus-infected cells, thereby aiding in viral clearance [2]. However, an exaggerated immune response orchestrated by these cells can lead to collateral tissue damage and exacerbate host injury. The innate immune response, while crucial for initial defense, can sometimes lead to unintended consequences. The surge in chemoattractant and cytokine production, commonly referred to as the “cytokine storm”, often exacerbates tissue damage and inflammation. What initially serves as a defense mechanism against an infection may inadvertently result in heightened immune-mediated pathology [3,4].

While studies have provided valuable insights into the mechanisms by which SARS-CoV-2 enters and functions within the body, our understanding of the host immune response remains incomplete. Despite significant progress in unraveling the pathophysiology of COVID-19, numerous questions persist regarding the intricacies of host-virus interactions and the immune responses elicited. As such, there is a critical need for further research to elucidate the complexities of the immune response to COVID-19 and its implications for disease progression and outcomes.

Among the array of immune cells comprising the first line of defense, eosinophils have received significant attention and scrutiny. This heightened focus can be attributed to their striking decrease in blood levels and their correlation with the disease severity [5,6]. Eosinophils contribute to the immune response by producing mediators such as cationic proteins. They also release cytokines involved in homeostasis and type 2 immune responses [7]. In COVID-19, there have been studies reporting low blood eosinophil levels following SARS-CoV-2 infection, and this eosinopenia was associated with greater disease severity and mortality [5,6,8,9,10,11]. The underlying reasons for this phenomenon are not entirely clear. However, in other inflammatory conditions such as allergic asthma, eosinophils are known to migrate to the airways following an allergic exposure, causing a transient decrease in peripheral levels and sustained increase in tissue eosinophils [12]. Therefore, we speculate that COVID-19 related eosinopenia may result from chemokine-mediated migration of eosinophils into the airways and tissue where they may be activated in response to the infection. The current study examined the changes in leukocyte levels, including eosinophils, and their production of mediators in patients infected with SARS-CoV2 to elucidate the underlying mechanisms driving immune dysregulation.

## 2. Results

### 2.1. Patient Demographics and Clinical Characteristics

A total of 84 COVID-19 positive and 65 COVID-19 negative control patients were enrolled. The 2 groups were overall matched for age and sex. The median age of participants was 62 years in the COVID-19 positive group and 64 years in the COVID-19 negative group (*p* = 0.430), 64.9% of COVID-19 positive patients and 46.4% of COVID-19 negative patients were non-smokers (*p* < 0.025) and prevalence of asthma and COPD was low (Table 1). At admission on day 0, 13.1% of COVID-19 positive patients and 15.9% of COVID-19 negative patients were receiving corticosteroids for treatment of pre-existing conditions, with the frequency increasing to 77.1% and 24.6%, respectively, during hospitalization. Among COVID-19 positive patients evaluated at day 1–10 visit, 25 were classified as mild, 45 moderate, and 14 severe disease.

### 2.2. Circulating Leukocyte Counts in COVID-19 Positive and COVID-19 Negative Control Patients

**Figure 1:**
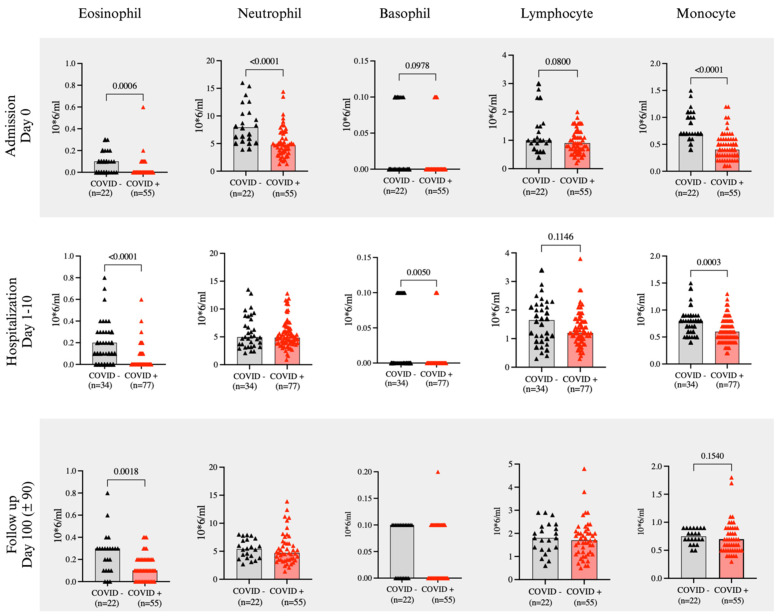
Circulating leukocyte levels. Comparison of leukocyte counts between COVID-19 positive (red) and COVID-19 negative (black) patients at admission (day 0), hospitalization (day 1–10) and follow up (day 100). Statistical testing was conducted using Mann-Whitney U test. Data are shown as individual points and median, and all significant differences (*p* < 0.05) are reported.

Leukocyte counts on CBC analysis for the 2 groups at three different time points are shown in Figure 1. Eosinophil levels were consistently lower in COVID-19 positive patients compared to control patients at all three time points (*p* < 0.05) (Figure 1). Monocytes and neutrophils were also significantly lower in COVID-19 positive patients during the admission visit, however at follow-up day 100, all white blood cell counts, except for eosinophils, were not significantly different from the control group (Figure 1). To further examine the contribution of steroid therapy, leukocyte counts were compared between groups stratified according to the presence and absence of steroid therapy (Figure S1), showing that in both patient groups, eosinophil levels were significantly lower with steroid treatment. Furthermore, among patients not receiving steroid treatment, the levels of eosinophils, neutrophils and monocytes remained significantly lower in COVID-19 positive patients (Figure S2). The significantly lower level of eosinophils in COVID-19 positive compared to COVID-19 negative patients was still present when patients were divided into groups based on asthma, smoking, and survival status. (*p* < 0.05) (Figure S3).

**Figure 2:**
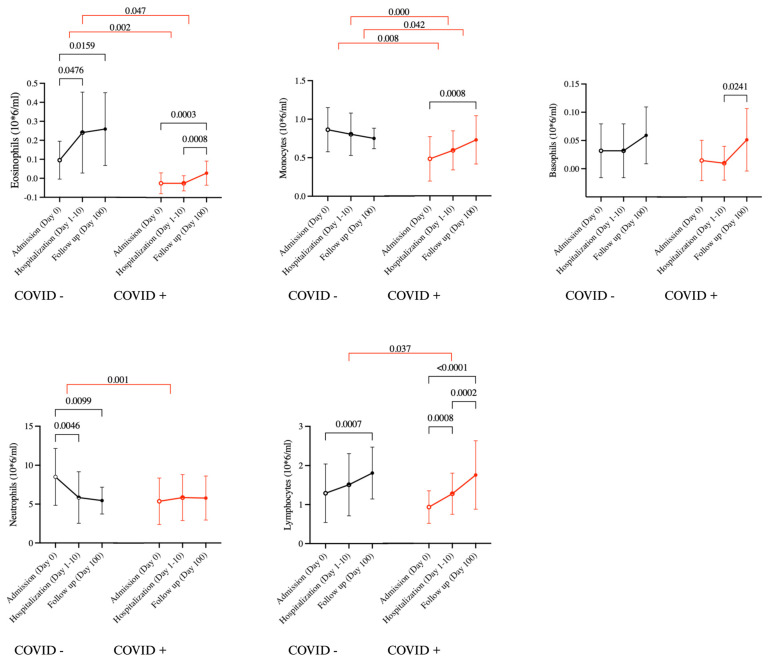
Kinetics of circulating leukocytes. Changes in the leukocyte count from admission (day 0) to the follow up (day 100) in COVID positive (red, n = 55) and COVID negative (black, n = 22) patients. Red lines indicate comparison between deltas. Statistical testing was conducted using paired *t* test (within each patient group) and independent *t* test (comparison of deltas between groups). All data are shown by mean (standard deviation), and all significant differences (*p* < 0.05) are reported.

Analysis of repeated measurements across the three timepoints showed that COVID-19 positive patients had a significant increase in the level of eosinophils, monocytes, basophils and lymphocytes over time. While in the COVID-19 negative group, there was a significant increase in eosinophils and lymphocytes but a decrease in neutrophils over time (*p* < 0.05) (Figure 2). Furthermore, the rate of eosinophil recovery from admission to follow up was slower in COVID-19 positive patients compared to COVID-19 negative controls (Figure 2).

### 2.3. Circulating Leukocyte Levels across Severities of COVID-19

**Figure 3:**
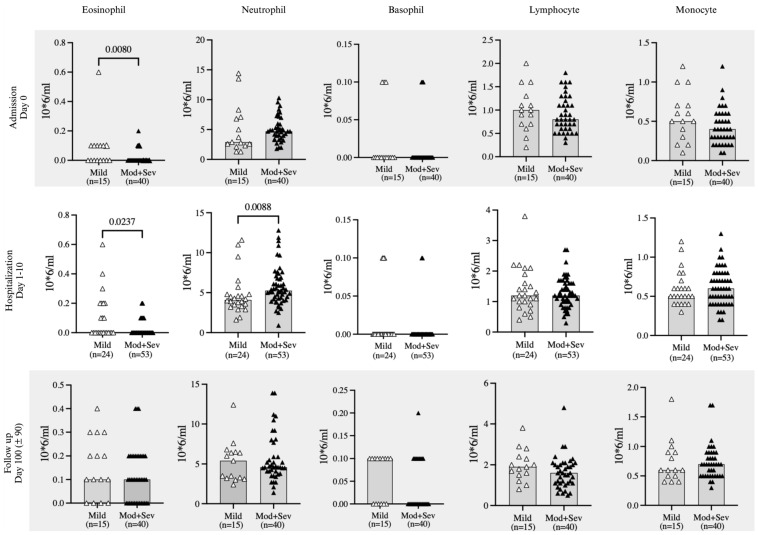
Circulating leukocytes stratified by severity of COVID-19. Comparison of leukocyte counts between mild (open symbols) versus moderate-severe (solid symbols) COVID-19 positive patients at admission, hospitalization and follow up. Statistical testing was conducted using Mann-Whitney U test. Data are shown as individual points and median, and all significant differences (*p* < 0.05) are reported.

Due to the small numbers of severe COVID-19 positive patients, the moderate and severe COVID-19 groups were combined to compare leukocyte levels between mild versus moderate-severe categories. Of note, while the numbers of COVID-19 positive patients receiving steroid therapy increased throughout admission, this was disproportionately higher for moderate-severe COVID-19 positive patients (Table 1). Eosinophil levels were consistently lower in the moderate-severe group compared to the mild group at admission (*p* = 0.008), and during hospitalization (*p* = 0.023). However, eosinophil levels were no longer different across severity groups at follow-up 100 days later (Figure 3). In contrast, neutrophil levels were higher in moderate-severe COVID-19 positive patients compared to those with mild symptoms, and this difference could reflect the higher frequency of steroid treatment in moderate and severe COVID19 groups (Table 1).

### 2.4. Serum Levels of Cytokines

**Figure 4:**
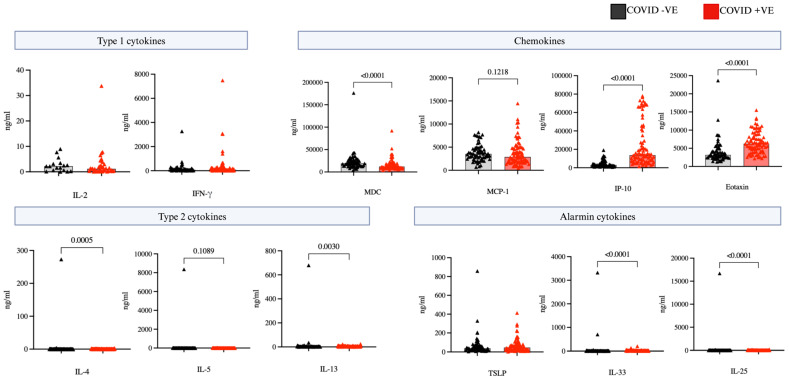
Circulating cytokine and chemokines. Comparison of circulating cytokine and chemokine levels between COVID positive (n = 84) and COVID negative (n = 54) patients during hospitalization (day 1–10). Statistical testing was conducted using Mann-Whitney U test, and all significant differences (*p* < 0.05) are reported.

**Figure 5:**
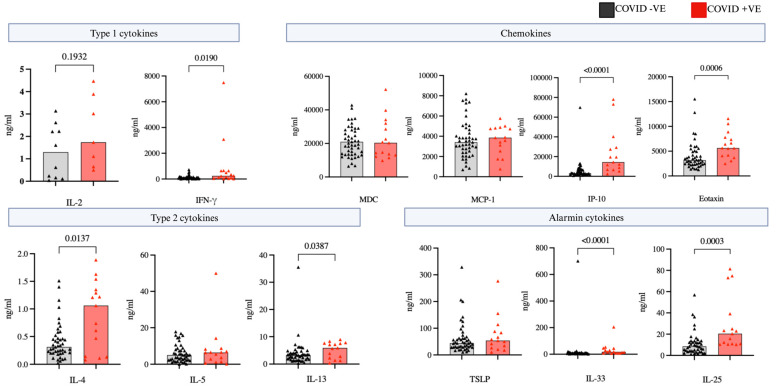
Circulating cytokines and chemokines in patients not administered steroid treatment. Comparison of circulating levels of cytokines and chemokines between COVID-19 positive and COVID-19 negative patients during hospitalization (day 1–10). No patients were being treated with corticosteroids at the time of sampling. Statistical testing was conducted using Mann-Whitney U test, and all significant differences (*p* < 0.05) are reported. Lower limit of detection in pg/mL (LLOD): IL-2 (0.09), IFN-γ (0.37), MDC (1.00), MCP-1 (0.16), IP-10 (0.12), eotaxin (0.2), IL-4 (0.02), IL-5 (0.14), IL-13 (0.24), TSLP (0.063), IL-33 (0.59), IL-25 (0.28).

During the hospitalization visit day 1–10, we examined serum levels of cytokines and chemokines to understand the shifts in circulating cells. Notably the level of eotaxin was significantly higher in the COVID-19 positive versus COVID-19 negative groups (Figure 4) and remained higher in the analysis of the non-steroid treated sub-groups (Figure S4). Type 2 cytokines IL-4 and IL-13 were also consistently higher in COVID-19 positive patients (*p* < 0.05), as well as the alarmin cytokines IL-33 and IL-25. While there were no notable disparities in type 1 cytokine levels including IL-2 and interferon gamma (IFN-γ) between the COVID-19 positive and COVID-19 negative groups, a focused analysis on individuals without steroid treatments revealed significantly elevated levels of IFN-γ in COVID-19 positive patients compared to COVID-19 negative. Eotaxin as well as IFN-γ–induced protein 10 (IP-10) levels were also significantly higher in steroid free COVID-19 patients compared to the negatives (Figure 5).

### 2.5. Serum Levels of Eosinophil Granule Proteins

**Figure 6:**
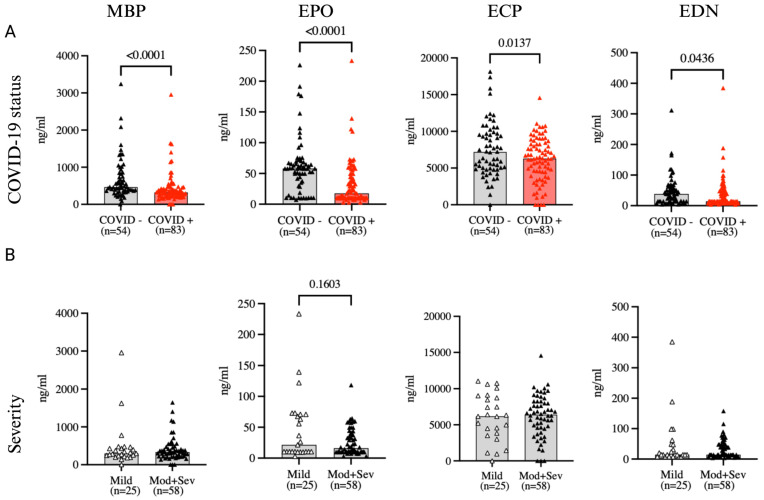
Circulating eosinophil granule proteins. Comparison of the level of circulating eosinophil granule proteins between (**A**) COVID-19 positive versus COVID-19 negative patients, and between (**B**) mild versus moderate-severe COVID-19 positive patients during hospitalization (days 1–10). All data are shown by median (range). Statistical testing was conducted using Mann-Whitney U test, and all significant differences (*p* < 0.05) are reported.

Elevated serum levels of eosinophil granule proteins have been described in the setting of activated eosinophils in tissue [13]. To assess whether migration to the lung might explain the decrease in blood eosinophils during COVID-19, serum levels of major basic protein (MBP), eosinophil peroxidase (EPO), eosinophil cationic protein (ECP), and eosinophil-derived neurotoxin (EPN) were measured during hospitalization day 1–10. Levels of all four eosinophil granule proteins were significantly lower in the COVID-positive patients compared to the COVID-19 negative group (all *p* < 0.05) (Figure 6). Significantly lower MBP and ECP levels were replicated in the sub-groups of patients not receiving steroids; numerically lower levels of EPO and EDN in COVID-19 positive patients did not meet statistical significance when comparing between these smaller sub-groups. (Figure S5). The levels of eosinophil granule proteins were not different between the mild and moderate-severe COVID-19 positive groups (*p* > 0.05) (Figure 6) but were weakly and positively correlated with circulating eosinophil counts (Table 2).

## 3. Discussion

Despite nearly five years having passed since its emergence, the complexities of COVID-19 continue to challenge scientific understanding. In this study, we observed a lower level of blood eosinophils in hospitalized COVID-19 positive patients compared to hospitalized COVID-19 negative patients. This downward trend was evident across multiple time points, including admission, hospitalization, and follow-up visits. These findings align with those of previous studies, which have also reported reduced eosinophil levels in COVID-19 patients [10,14,15].

Although the reason for lower eosinophil levels is not completely clear, one plausible explanation is that the reduced eosinophil count in the blood reflects their migration to the tissue where the infection is most active. Mature eosinophils typically circulate in the bloodstream and migrate to the site of inflammation in response to chemoattractants and inflammatory mediators, such as eotaxin. This migration might explain the observed eosinopenia in COVID-19, contrasting with the eosinophilia seen in other respiratory infections such as influenza and respiratory syncytial virus. [12,16]. In our study, we observed higher levels of eotaxin in the serum of COVID-19 patients compared to non-COVID-19 patients. Eotaxin is known to be released by activated epithelial cells in response to viral invasion [17]. Additionally, eotaxin has been shown to be elevated in other inflammatory conditions of the airways, such as asthma. Studies investigating serum and plasma eotaxin levels in asthmatic patients have demonstrated a positive correlation between higher eotaxin levels and more severe and impaired lung function [18,19,20]. Higher eotaxin levels have also been reported in other viral respiratory infections such as influenza and common cold [21,22]. Therefore, it is reasonable to speculate that the elevated eotaxin levels observed in COVID-19 patients may contribute to the migration of eosinophils to the airway tissue, where the infection is primarily active. This phenomenon, in turn, could serve as a biomarker of worse lung impairment and respiratory distress in COVID-19 patients.

Existing literature consistently indicates a positive prognosis when eosinophil levels are elevated. For instance, Zein et al. reported improved COVID-19 outcomes in patients with eosinophilia [23]. Similarly, Ferastraoaru et al. demonstrated that hospitalized COVID-19 patients with asthma and eosinophil counts of ≥150 cells/μL had a lower likelihood of succumbing to COVID-19 complications [24]. Our observations align with these findings, demonstrating that patients with more severe clinical disease showed lower eosinophil counts and higher needs for oxygen therapy. This negative relationship between eosinophils and disease severity may shed light on why asthmatic patients appear to be less susceptible to COVID-19, with a significantly lower prevalence of COVID-19 among individuals with asthma [25,26,27]. These findings underscore the potential utility of eosinophil levels as a prognostic marker for COVID-19 outcomes [28,29,30].

After observing the low number of eosinophils in the blood of COVID-19 patients, we hypothesized that these eosinophils may have migrated to the tissue, thus explaining their absence in the bloodstream. The elevated levels of eotaxin further support a potential mechanism for attracting these cells to the affected tissues as well. To investigate further, we measured the levels of eosinophil granule proteins in the serum, expecting to find higher levels indicative of eosinophil activity in tissue. Contrary to our expectations, we observed lower levels of MBP, ECP, EDN and EPO in the serum of COVID-19 positive patients compared to COVID-19 negatives.

One potential factor contributing to the lower eosinophil levels observed in COVID-19 patients during hospitalization was the increased number of patients receiving steroid treatment. Upon admission to the hospital, approximately 13 percent of COVID-19 patients were already receiving steroid therapy, a percentage that dramatically increased to 77.1 percent during hospitalization. Corticosteroids are well-known for their ability to reduce eosinophil levels through a number of mechanisms including by reducing levels of eosinophil growth factors and augmenting the transcription of genes associated with apoptosis. Research has demonstrated that the glucocorticosteroid receptor complex effectively hampers the function of transcription factors responsible for growth (i.e., AP-1) by forming complexes with them [20,31,32,33,34]. Corticosteroid treatment is also known to inhibit the release of eosinophil granule proteins [18], which could account for some of the reduction of these proteins observed in the COVID-19 patients [35]. Alternatively, steroid treatment has been demonstrated to prompt the migration of eosinophils back to the bone marrow and induce their apoptosis [36]. However, when analyzing the eosinophil levels in COVID-19 patients who were not receiving steroids, we still observed low eosinophil levels, suggesting that factors beyond steroid treatment may be influencing eosinophil dynamics in COVID-19 infection. The absence of eosinophils was reported by studies on post-mortem lung autopsies, in the early days of COVID-19 [37,38]. In contrast to our data showing a reduction in eosinophil degranulation products in COVID-19 patients, a study published on fatal cases of COVID-19 in patients predominantly receiving glucocorticoid therapy, revealed upregulation of the *EDN* gene in lung tissue eosinophils. This suggests that despite the reduced numbers of eosinophils in the tissue, these cells may still be releasing their protein compounds. However, due to their diminished presence in the tissue, their functional impact may be limited [39]. Therefore, while our findings provide valuable insights into circulating eosinophil granule protein levels, further investigation is needed to elucidate the tissue-specific dynamics of these proteins in COVID-19 patients.

The administration of steroids largely contributes to the observed increase in neutrophil levels during hospitalization visits. During admission, when fewer patients were using steroid therapy, COVID-19-positive individuals exhibited lower neutrophil counts, suggesting a potential cytopenic effect of COVID-19. With the addition of steroid treatment, neutrophil levels also rose and normalized compared to non-COVID-19 patients. Corticosteroids are commonly recognized as immunosuppressive agents that induce apoptosis of white blood cells, however glucocorticoids such as dexamethasone induce anti-apoptotic effects on neutrophils and retention of neutrophils in the circulation [40,41]. In our sub analysis across COVID-19 severity, we observed a notable increase in neutrophil levels among moderate-severe COVID-19 patients in contrast to those with milder cases, aligning with the findings reported by Ryan et al., who also observed elevated neutrophil counts in severe COVID-19 cases [42].

Among other leukocytes, monocytes were significantly lower in COVID-19 patients during admission and after hospitalization. Monocytes play a pivotal role in cytokine storm formation in COVID-19 patients, as they produce high levels of pro-inflammatory cytokines such as IL-6 and TNF-α, contributing to an exaggerated immune response to the infection. Additionally, monocytes as well as basophils and eosinophils, have been reported to express the ACE2 receptor, which is necessary for viral entry, and studies have demonstrated their susceptibility to infection [43,44]. This dual role of monocytes in both immune response amplification and viral susceptibility may explain the observed reduction in monocyte levels in the blood of COVID-19 patients. When examining the changes in leukocyte levels from admission to hospitalization and follow-up stages, we saw a consistent trend that all cell counts increase from admission to follow-up, paralleling the process of recovery. During recovery, leukocyte numbers returned to baseline, although remaining slightly lower compared to those observed in COVID-19 negative patients. Extending the follow-up period beyond 100 days would have provided more insight into the duration needed for leukocyte levels to fully normalize.

In our last set of experiments, we examined cytokine measurements in the serum as indicators of immune responses. In all patients we observed that cytokines associated with a type 1 immune response did not significantly differ between COVID-19-positive and negative patients. However, when restricting the analysis to patients not administered steroid treatment we observed higher levels of interferon-gamma in COVID-19-positive patients compared to COVID-19-negative (*p* = 0.019), with no significant difference in IL-2 levels. This lack of difference in IL-2 levels could potentially be attributed to the levels of this cytokine being close to the limit of detection, reducing our ability to detect a difference between groups. These higher levels of interferon-gamma are consistent with other reports indicating elevated type 1 cytokine levels in COVID-19 patients as a consequence of infection [45]. Measurements of cytokines associated with a type 2 immune response demonstrated higher levels of IL-13 and IL-4 in the COVID-19-positive group compared to the negative group. We also observed higher levels of the alarmin cytokines IL-33 and IL-25 in COVID-19-positive patients, which could be driving type 2 inflammation through polarization of T helper cells or activation of ILC2 [46]. Paula et al. reported higher IL-4 levels and correlated tissue damage resulting from the heightened type 2 response. This cascade of cytokine signaling underscores the complex interplay between immune mediators in the context of COVID-19 infection [47].

Analysis of cytokine and chemokine levels in patients who did not undergo dexamethasone treatment demonstrated elevated levels of eotaxin and IFN-γ–induced protein 10 (IP-10) in COVID-19-positive patients. The elevation of IP-10 has been consistently reported in COVID-19 patients and is correlated with disease severity and progression [48,49]. The higher eotaxin levels observed in COVID-19 patients may be explained by activated airway epithelium and leading to potent chemoattraction of eosinophil migration to the airway tissue thereby reducing eosinophil levels in bloodstream. Genome wide association studies (GWAS) have identified regions (risk haplotypes) in the chromosome which are significantly associated with COVID-19 severity in patients [50,51]. This region is located on chromosome 3p21.31 and includes chemokine receptor genes including CCR3, the receptor for eotaxin. The presence of CCR3 in this risk haplotype suggests an association between eotaxin signaling and risk of COVID-19 severity.

There are some limitations to our study. Firstly, due to its observational nature, we cannot establish causality between observed immune response patterns and COVID-19 outcomes. Additionally, our reliance on serum samples for cytokine analysis may not fully reflect tissue-specific immune responses. The approximate 3 month follow up of longitudinal data limits our understanding of immune cell changes over a longer time course of COVID-19 infection and recovery. Lastly, our study of cell mediators was confined to blood samples collected at a single hospitalization timepoint, restricting our ability to examine immune response kinetics. Despite these limitations, our study provides valuable insights into COVID-19 immune dynamics, paving the way for future research.

In conclusion, our study demonstrated reduced levels of eosinophils and their degranulation products in COVID-19 patients, despite heightened immunity demands, emphasizing the complexity of immune dynamics. Further investigation into tissue-specific responses and therapeutic impacts, like steroid treatment, is crucial for optimizing COVID-19 management strategies.

## 4. Materials and Methods

### 4.1. Study Participants and Recruitment

Between February and October 2021, patients confirmed positive for COVID-19 by real-time reverse-transcription-PCR (COVID-19 positive) on nasopharyngeal swabs were recruited upon hospital admission. Age and sex-matched hospitalized patients, confirmed negative for COVID-19 by PCR and did not exhibit any signs of respiratory infection, were recruited as controls. The study was approved by the Hamilton Integrated Research Ethics Committee and all patients provided written inform consent.

### 4.2. Demographics and Clinical Characteristics

Demographic information and clinical characteristics were extracted from electronic medical records. Blood samples were collected for complete blood counts (CBC) at three timepoints: on admission at the time of positive COVID-19 test (day 0), within 10 days after admission (day 1–10), and at day 100 after admission during follow up. A blood sample was also collected from each patient at day 1–10 for mediator analysis.

COVID-19 positive patients were classified according to disease severity using a classification described by the food and drug administration [52] Accordingly, patients hospitalized but not needing supplemental oxygen were categorized as mild disease; while those requiring oxygen therapy were considered moderate, and the severe group included patients needing non-invasive and invasive ventilatory support. Patients were assigned to one of these categories based on these criteria assessed in the first 10 days of admission.

### 4.3. Mesoplex Assay

Serum samples were analyzed for 17 analytes consisting of type 1, type 2, and alarmin cytokines, as well as chemoattractant levels using an ultra-sensitive human cytokine multiplex electrochemiluminescence-based immunoassay (Meso Scale Discovery, Gaithersburg, MD, USA). The assay was conducted on the MESO QuickPlex SQ 120MM instrument (MSD) according to the manufacturer’s protocol. Protein levels were quantified in picograms per milliliter of blood (pg/mL).

### 4.4. Luminex Assay

A multiplex assay was used to measure four eosinophil granule proteins (EGP); major basic protein (MBP), eosinophil-derived neurotoxin (EDN), eosinophil cationic protein (ECP), and eosinophil peroxidase (EPO) in de-identified serum. To prevent aggregation, the samples underwent reduction and alkylation. Purified EGP standards were diluted in assay buffer (consisting of 1× phosphate-buffered saline (PBS), 1% bovine serum albumin (BSA), and 0.05% Tween-20) to an initial concentration of 500 ng/mL each, followed by serial dilution at a ratio of 1:3. Patient samples were diluted 1:220 in assay buffer. The assay results were determined as concentrations derived from the standard curve using mean fluorescent intensity (MFI) from duplicate samples.

### 4.5. Statistical Analysis

The data are presented as median and range. A significance level (alpha) of 0.05 was used for statistical analysis. Data analysis was conducted using IBM SPSS Advanced Statistics (IBM Corp., Version 24.0, Armonk, NY, USA) and GraphPad Prism software version 9.0 (GraphPad Software, San Diego, CA, USA). Nonparametric Mann-Whitney U test for data not normally distributed, were performed to assess differences between groups.

## Figures and Tables

**Table 1:** Patient demographics and clinical characteristics. COVID-19 positive and negative patients shown as number of patients (percentage of patients). *p* values comparing all COVID-19 positive and COVID-19 negative patients were calculated using Mann-Whitney U test for continuous variables and Fisher’s exact test for categorical variables. Bold font indicates significant difference (*p* < 0.05). * Data shown as median (interquartile range).

		COVID-19 Positive				COVID-19 Negative	*p* Value
		Mild = 25 n (%)	Moderate = 45 n (%)	Severe = 14 n (%)	All = 84 n (%)	All = 65 n (%)	
Demographics							
Sex	Male	13 (52)	26 (57.8)	7 (50)	46 (54.8)	32 (49.3)	0.501
	Female	12 (48)	19 (42.2)	7 (50)	38 (45.2)	33 (50.7)	
Age	Years *	66 (34)	60 (28)	58 (37)	62 (29.75)	64 (18)	0.430
Steroid treatment	Admission	2 (8)	6 (13.3)	3 (21.4)	11 (13.1)	11 (15.9)	0.649
	Hospitalization	8 (32)	39 (86.7)	7 (50)	54 (77.1)	16 (24.6)	**0.00**
Smoking Status	Never	14 (58.3)	29 (67.4)	7 (70)	50 (64.9)	26 (46.4)	**0.025**
	Ex-smoker	6 (25)	2 (4.7)	0 (0)	19 (24.7)	18 (32.1)	
	Current Smoker	4 (16.7)	12 (27.9)	3 (30)	8 (10.4)	12 (21.4)	
Comorbidities							
Asthma	N (%)	2 (8)	4 (8.9)	2 (14.3)	8 (9.5)	7 (10.1)	0.677
COPD	N (%)	4 (16)	3 (6.7)	1 (7.1)	8 (9.5)	11 (15.9)	0.231
Diabetes	N (%)	3 (12)	2 (4.4)	1 (7.1)	0 (0)	1 (1.4)	0.268

**Table 2:** Circulating eosinophils and eosinophil granule proteins. Relationship between blood eosinophil counts and their granule proteins in all COVID-19 positive patients, and in COVID-19 positive patients who were not treated with steroids. Statistical testing was conducted using Pearson correlation test. Statistical significance at *p* < 0.05.

Variable	COVID-19 Positive (n = 83)	Steroid-Free COVID-19 Positive (n = 16)
	Pearson r (*p*-value)	Pearson r (*p*-value)
MBP	0.28 (0.01)	0.24 (0.36)
EPO	0.24 (0.03)	0.28 (0.29)
EDN	0.20 (0.08)	0.07 (0.78)
ECP	0.09 (0.42)	0.51 (0.04)

## Data Availability

Data is unavailable due to privacy restrictions.

## Supplementary Materials

The following supporting information can be downloaded at: https://www.mdpi.com/article/10.3390/ijms25126427/s1.
