# Supplementary material for: Immune Response Dynamics and Biomarkers in COVID-19 Patients
Source: Int J Mol Sci. 2024 Jun 11;25(12):6427. doi: 10.3390/ijms25126427 (PMC11204302; doi:10.3390/ijms25126427)
Supplement: Supplementary file 1 [file ijms-25-06427-s001.zip › ijms-3036630-supplementary.pdf]

Supplementary Materials

Contents

Supplementary Figure 1. Effect of corticosteroid treatment on circulating leukocyte counts. ... 2

Supplementary Figure 2. Circulating leukocyte levels in patients not treated with steroids. 3

Supplementary Figure 3. Circulating eosinophil levels stratified by demographic status. 4

Supplementary Figure 4. Circulating eosinophil granule proteins in patients not administered steroid treatment. 5

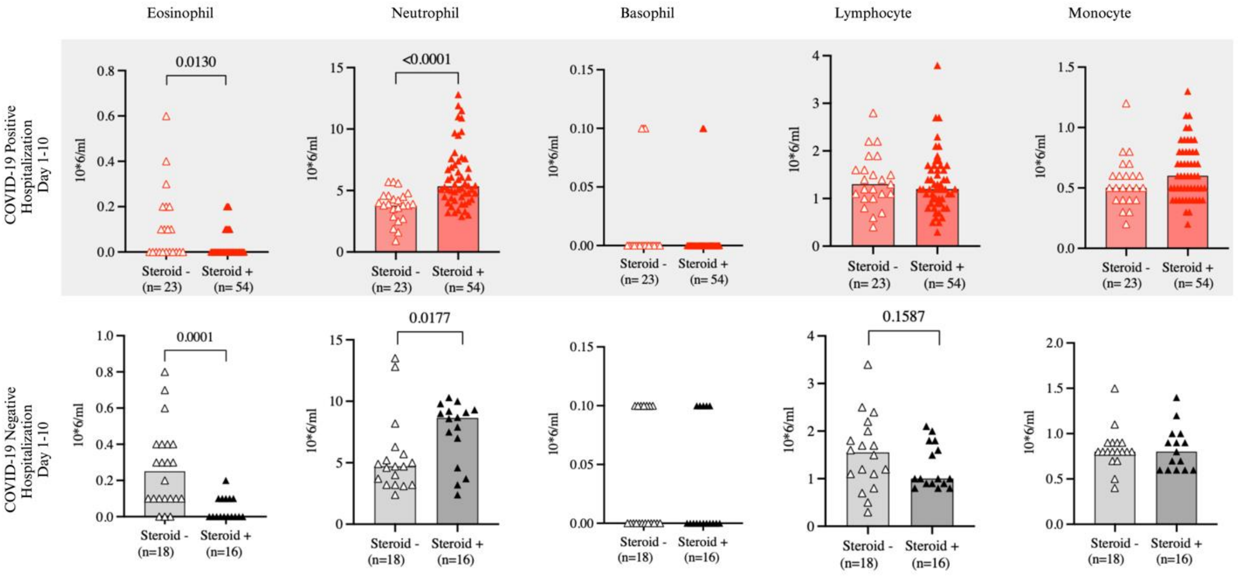

Supplementary Figure 1. Effect of corticosteroid treatment on circulating leukocyte counts. Comparison of leukocyte counts in COVID-19 positive (red) and COVID-19 negative (black) patients at the hospitalization (day 1-10). Statistical testing was conducted using independent T test and its non-parametric equivalent. Data are shown as individual points and median, and all significant differences ($P < 0.05$) are reported.

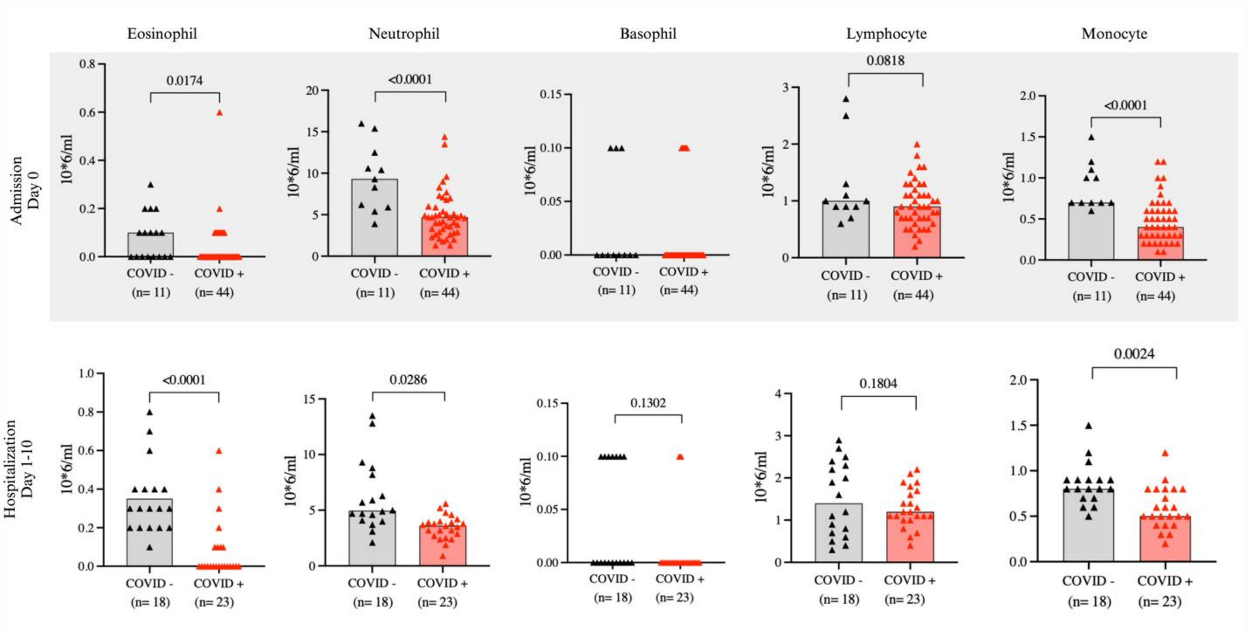

Supplementary Figure 2. Circulating leukocyte levels in patients not treated with steroids. Comparison of leukocyte counts between COVID positive (red) and COVID negative (black) patients not using steroid treatment at admission (day 0) and hospitalization (day 1-10). Statistical testing was conducted using Mann-Whitney U test. Data are shown as individual points and median, and all significant differences ($P < 0.05$) are reported.

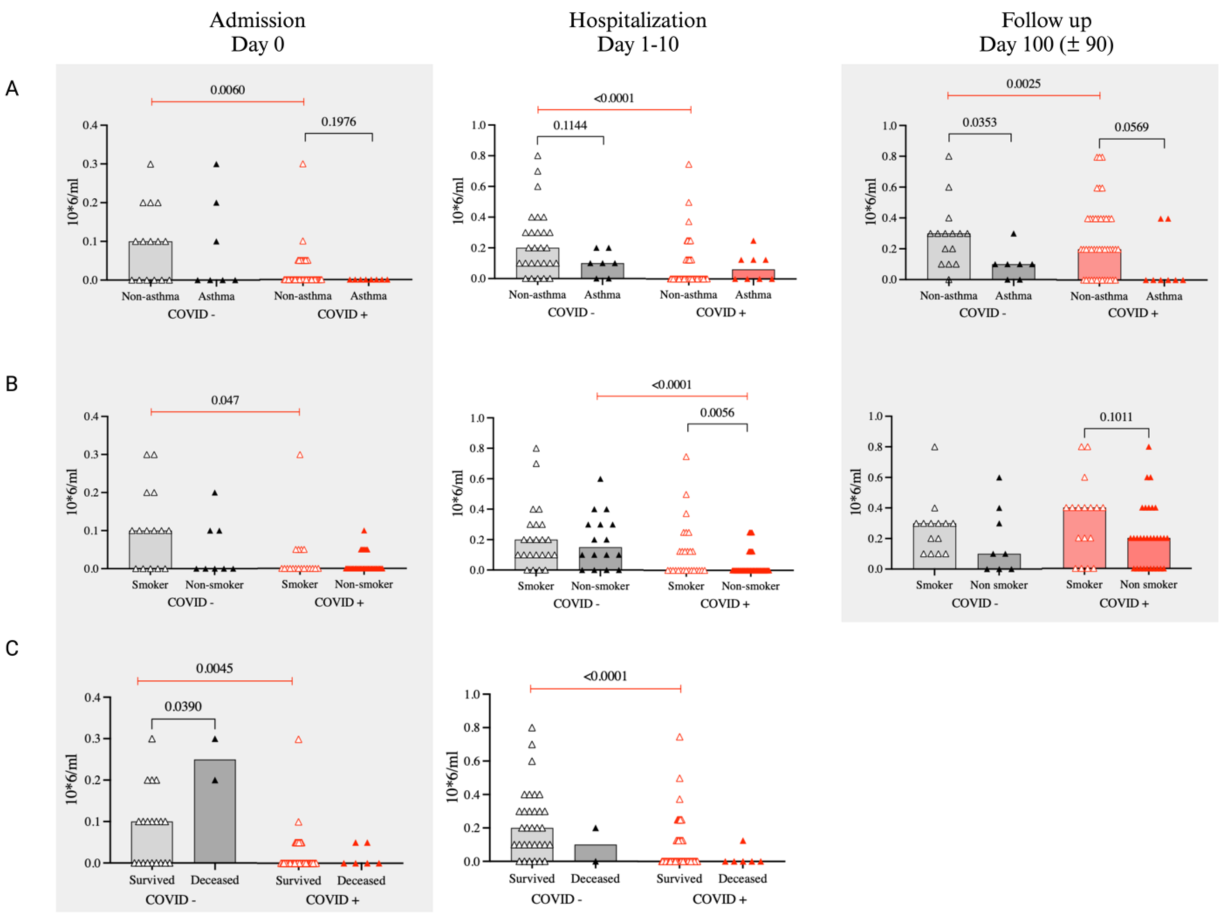

Supplementary Figure 3. Circulating eosinophil levels stratified by demographic status. Comparison of eosinophil counts between COVID-19 positive (red) and COVID-19 negative (black) patients divided into sub-groups of A) asthmatic versus non-asthmatic, B) smokers versus no-smokers, and C) survived versus deceased patients, measured at admission, and hospitalization and follow up. Statistical testing was conducted using Mann-Whitney U test. Data are shown as individual points and median, and significant differences ($P < 0.05$) are reported within COVID-19 positive and COVID-19 negative sub-groups (black line) and between COVID-19 positive and COVID-19 negative sub-groups (red line).

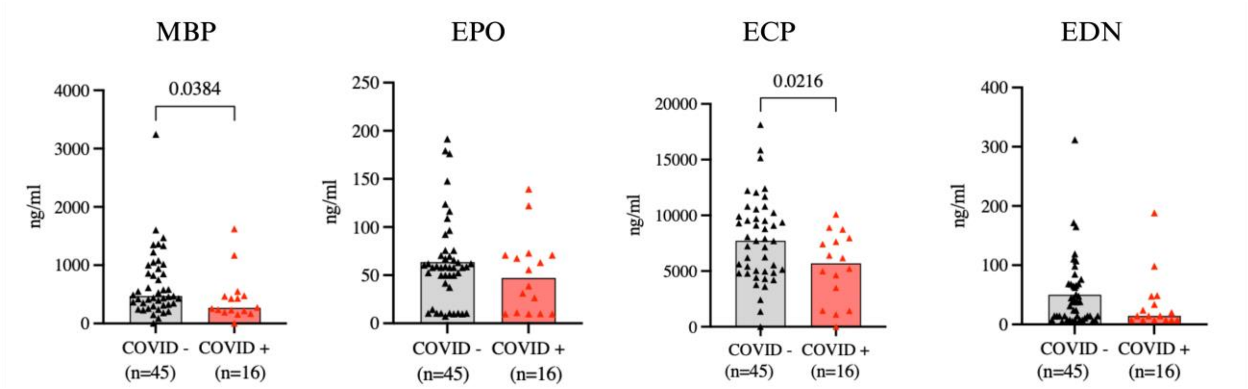

Supplementary Figure 4. Circulating eosinophil granule proteins in patients not administered steroid treatment. Comparison of the level of circulating eosinophil granule proteins between COVID-19 positive and COVID-19 negative patients not receiving steroid treatment. All data are shown by median (range). Statistical testing was conducted using Mann-Whitney U test, and all significant differences ($P < 0.05$) are reported.
